# A Case of *Fusobacterium necrophorum* without Lemierre's Syndrome Mimicking Acute Leptospirosis

**DOI:** 10.1155/2019/4380429

**Published:** 2019-09-22

**Authors:** Ryo Yasuhara, Shunichi Shibazaki, Takayoshi Yamanouchi

**Affiliations:** ^1^Tokyo Medical and Dental University Hospital Faculty of Medicine, Tokyo, Japan; ^2^Department of Emergency and General Internal Medicine, Hitachinaka General Hospital, Hitachinaka, Japan; ^3^Department of Cardiology, Hitachinaka General Hospital, Hitachinaka, Japan

## Abstract

Jaundice, conjunctival hyperemia, and acute kidney injury (AKI) are the characteristics of leptospirosis. However, it is not well known that *Fusobacterium necrophorum* infection can have a clinical picture similar to that of leptospirosis. A 38-year-old man was admitted with jaundice, conjunctival hyperemia, and AKI for 7 days. Chest CT scan showed multiple pulmonary nodules, atypical for leptospirosis. We started treatment with IV piperacillin-tazobactam and minocycline. He became anuric and was urgently started on hemodialysis on the second hospital day. Later on, blood cultures grew *Fusobacterium necrophorum* and other anaerobic bacteria. Antibody and PCR assays for *Leptospira* were negative. We narrowed the antibiotics to IV ceftriaxone and metronidazole. He responded well to the treatment and was discharged on the 18th hospital day. *F. necrophorum* infection is known to cause mixed infection with other anaerobic bacteria. The resistance of many anaerobic bacteria continues to progress, and *F. necrophorum* itself sometimes produces *β*-lactamase. This case highlights the potential risks of using penicillin before diagnosis of leptospirosis.

## 1. Introduction

Jaundice, conjunctival hyperemia, and acute kidney injury (AKI) are the characteristics of leptospirosis [1, 2], which can be treated with penicillin [3]. However, *Fusobacterium necrophorum* infection may mimic leptospirosis [4–6], which is not well known. *F. necrophorum* often causes mixed infections with other anaerobic bacteria. In addition, the antimicrobial resistance of anaerobic bacteria is serious, and sometimes *F. necrophorum* itself also produces *β*-lactamase [7]. We present a case of *F. necrophorum* mixed-infection mimicking leptospirosis that might have treatment failure with penicillin.

## 2. Case Summary

A 38-year-old man presented with a seven-day history of fatigue, fever, and jaundice. He initially went to a nearby clinic as symptoms worsened. Subsequently, he was also diagnosed with AKI and transferred to our hospital. He had no contact with rats and did not do field work. He had no prior medical history.

Vital signs at the time of visit were as follows: blood pressure 116/73 mmHg, pulse 110/min, body temperature 37.4°C, and respiration rate 18/min. His ocular conjunctivae wereyellow-tinged and congested. His oral cavity was not swollen or reddish; however, he complained of a sore throat.

**Figure 1:**
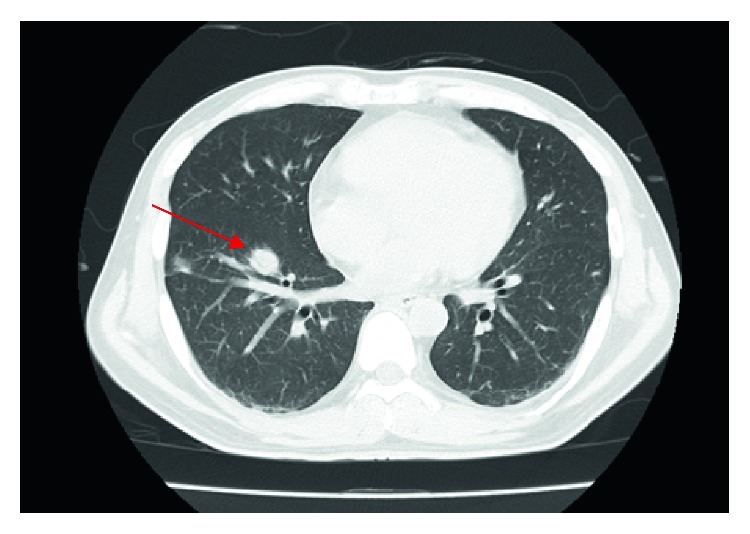
The chest computed tomography on the 1^st^ day showing multiple nodules (red arrow).

Blood tests revealed a white blood cell count of 18900/*μ*L. His hemoglobin was 10.8 g/dL and platelet count was 2.6 × 10^4^/*μ*L, and no schizocytes were observed. Aspartate aminotransferase was 31 U/L, alanine aminotransferase 18 U/L, lactate dehydrogenase 234 U/L, alkaline phosphatase 340 U/L, *γ*-glutamyltransferase 41 U/L, total bilirubin 12.4 mg/dL, and direct bilirubin 10.5 mg/dL, which is above the threshold for hyperbilirubinemia. Blood urea nitrogen was 85.6 mg/dL, creatinine was 8.3 mg/dL, and C-reactive protein was elevated to 29.26 mg/dL (normal range 0.0–0.3 mg/dL). In urine qualitative assays, positive results were observed for proteins, urobilinogen, bilirubin, and occult blood. In addition, urine sugar was positive despite not having diabetes. Chest CT showed multiple nodules in both lungs (Figure 1). Abdominal CT showed no abnormality that could explain the jaundice and kidney failure.

We initially strongly suspected leptospirosis. However, other infectious diseases could not be excluded because the multiple nodules in the lungs were atypical for leptospirosis.

**Figure 2:**
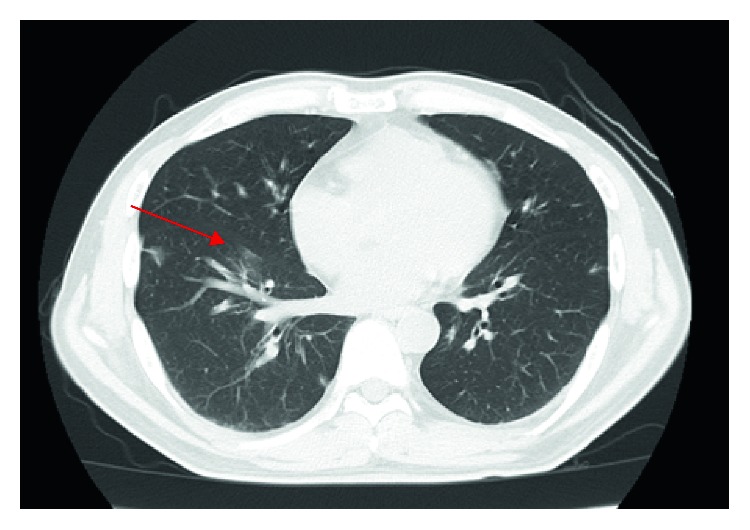
The chest computed tomography on the 38th day showing multiple nodules disappeared (red arrow).

**Figure 3:**
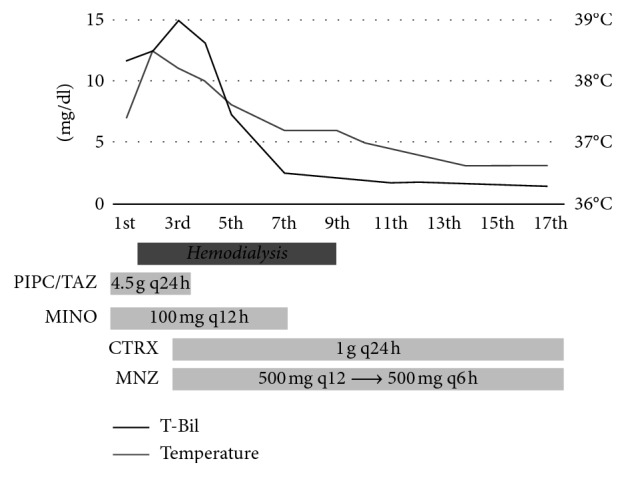
The clinical course. T-Bil: total bilirubin; PIPC/TAZ: piperacillin-tazobactam; MINO: minocycline; CTRX: ceftriaxone; MNZ: metronidazole.

Because there was a possibility of various diseases, we avoided IV penicillin and started IV piperacillin-tazobactam and minocycline instead. He became anuric and was started on urgent hemodialysis on the second hospital day. Later on, antibody and PCR assays for *Leptospira* were negative. *F. necrophorum* was isolated in blood cultures on the second day. Further late, *Streptococcus intermedius* were isolated in blood cultures on the seventh day. We diagnosed his main disease as *F. necrophorum* rather than *S. intermedius* bacteremia based on the past case reports and the speed of positive blood cultures. Since *F. necrophorum* is famous for Lemierre's syndrome, we performed ultrasound in his jugular vein but found no thrombus. In addition, the dentist diagnosed a minor periodontal infection. *F. necrophorum* is thought to spread from periodontal infections. The main pathogen was *F. necrophorum*, but other anaerobic bacteria were also suspected to be involved. In addition, we were not able to test the susceptibility of *F. necrophorum* to penicillin. His treatment was thus de-escalated to IV ceftriaxone (CTRX) 1 g every 24 h and metronidazole (MNZ) 500 mg every 12 h on the 3rd day. Later, MNZ was increased up to 500 mg every 6 h. These treatments were continued up to the 17th day. The multiple lung nodules formed cavities and later disappeared after definitive therapy (Figure 2). He responded well to the treatment, was taken off hemodialysis, and discharged on the 18th hospital day. After discharge, he continued the treatment with amoxicillin and MNZ orally for three weeks (Figure 3). He had no relapse after three months of follow-up.

## 3. Discussion

This was a case of *F. necrophorum* infection without Lemierre's mimicking leptospirosis. Differentiating between these two diseases is important from the viewpoint of early antibacterial drug selection and identification of complications.

Leptospirosis is a zoonosis caused by the pathogenic spirochete *Leptospira* spp. Its occurrence is distributed throughout the world but is especially frequent in tropical areas [8]. The main vectors are rodents. *Leptospira* infects humans via contact of the skin, mucous membranes, and conjunctiva with animal urine and contaminated water and soil. After the incubation period, which is usually ten days, fever, muscle pain, and headache appear in 75–100% of patients [9]. When leptospirosis becomes severe, it complicates with symptoms of jaundice and renal failure [1]. That is also known as Weil's disease. Conjunctival hyperemia has also been observed in 55% of cases and is a symptom useful for differentiation from other infectious diseases [2].

*F. necrophorum* is an anaerobic Gram-negative bacillus that is resident in the oral cavity [10]. *F. necrophorum* infection decreased after the spread of penicillin [11]; however, *F. necrophorum* infection, and especially pharyngitis, has increased in recent years [12]. In addition, *F. necrophorum* infection has a 5–9% mortality rate even with antibiotics [10].

*F. necrophorum* infection is sometimes accompanied by jaundice. Especially, most cases of frank jaundice or hyperbilirubinemia due to *F. necrophorum* infection had a thrombus in the internal jugular vein, which progressed to Lemierre's syndrome [13]. This is a new point from this case: this case had no thrombus in the internal jugular vein. The exact frequency of jaundice by *F. necrophorum* is unknown. Moreover, there are completely different reports: frank jaundice is rare [14] or up to 49% [15]. In addition, the exact mechanism of jaundice by *F. necrophorum* remains unclear. We assume the lipopolysaccharide of *F. necrophorum* may affect jaundice because hyperbilirubinemia in sepsis is due to cholestasis by the lipopolysaccharide of bacteria [16], and the main toxicity of *F. necrophorum* is also from its lipopolysaccharide [17]. In addition, hemophagocytic lymphohistiocytosis can also be a differential diagnosis of jaundice and liver damage. In general, diagnosis of hemophagocytic lymphohistiocytosis in adults is due to multiple findings: fever ≥38.5°C, splenomegaly, pancytopenia, hypertriglyceridemia, and hyperferritinemia [18]. In this case, we did not measure triglyceride and ferritin, nor do a bone marrow puncture; therefore, we cannot deny hemophagocytic lymphohistiocytosis completely.

*F. necrophorum* sometimes has remote infection sites; in particular, septic embolism in the lung is found in 97% of *F. necrophorum* infections [13]. Septic embolism in the lung is an important feature of *F. necrophorum* infection and is not usually seen in leptospirosis. As the presence or absence of septic embolism can also affect the treatment period, it is important to differentiate between the two diseases. Incidentally, in this case, septic embolism in the lung was observed, and it was thought to be a disease condition close to Lemierre's syndrome, which is a severe clinical disease type of *F. necrophorum* infection characterized by pharyngitis and purulent thrombophlebitis in the jugular vein accompanied with bacteremia. However, thrombophlebitis of the jugular vein was not found by ultrasound or CT scan in this case.

This case showed the potential risks of using penicillin before diagnosis of leptospirosis. Penicillin is common for the treatment of leptospirosis [3]. *F. necrophorum* infection often causes a mixed infection with other anaerobic bacteria. In fact, our case was a mixed infection with *S. intermedius*. Antimicrobial resistance of anaerobic bacteria continues to progress; some anaerobic bacteria is resistance to penicillin. In addition, *F. necrophorum* itself occasionally produce *β*-lactamase [7]; that is, 2% of *F. necrophorum* is resistance to penicillin [19]. Unfortunately, we did not test susceptibility of *F. necrophorum* to PCG. Therefore, it is not exactly known whether this case has failed with penicillin or not; however, it remains a possibility that it may fail in a case of *F. necrophorum* and mixed infection. *F. necrophorum* is sensitive to CTRX and *β*-lactamase inhibitor combination drugs [2], and CTRX is equivalent to the penicillin group in the therapeutic effect against *Leptospira* [20, 21]. Therefore, initial treatment with cephalosporin or a *β*-lactamase inhibitor combination drug may be preferred when the cause of an infection has not been or cannot be distinguished.

We experienced a case of *F. necrophorum* infection mimicking leptospirosis. This case suggests the potential risks of using penicillin before a diagnosis of leptospirosis.
